# Non-Inferiority of Sutureless Aortic Valve Replacement in the TAVR Era: David versus Goliath

**DOI:** 10.3390/life12070979

**Published:** 2022-06-29

**Authors:** Alina Zubarevich, Marcin Szczechowicz, Lukman Amanov, Arian Arjomandi Rad, Anja Osswald, Saeed Torabi, Arjang Ruhparwar, Alexander Weymann

**Affiliations:** 1 Department of Thoracic and Cardiovascular Surgery, West German Heart and Vascular Center, University of Duisburg-Essen, 45117 Essen, Germany; marcin.szczechowicz@yahoo.com (M.S.); lukman.amanov@uk-essen.de (L.A.); anja.osswald@uk-essen.de (A.O.); arjang.ruhparwar@uk-essen.de (A.R.); weymann.alexander@googlemail.com (A.W.); 2 Department of Medicine, Faculty of Medicine, Imperial College London, London SW7 2AZ, UK; arian.arjomandi-rad16@imperial.ac.uk; 3 Department of Anesthesiology, University Hospital Cologne, 50931 Cologne, Germany; saeed.torabi@gmail.com

**Keywords:** SU-AVR, TA-TAVR, sutureless aortic valve, Perceval

## Abstract

Background: The rapid development of transcatheter treatment methods has made transcatheter aortic valve replacement (TAVR) a feasible alternative to conventional surgical aortic valve replacement (SAVR). Recently, indications for TAVR have been expanded to intermediate- and low-risk patients, although there still remains a portion of ineligible patients. We sought to evaluate and compare our experience with sutureless SAVR and transapical TAVR in the “grey-area” of patients unsuitable for transfemoral access. Methods: Between April 2018 and June 2021, 248 consecutive patients underwent a sutureless SAVR (SU-SAVR) or TA-TAVR at our institution. We performed a pair-matched analysis and identified 56 patient pairs based on the EuroSCORE II. All transcatheter procedures were performed using SAPIEN XT/3™ prostheses, while all surgical procedures deployed the Perceval (LivaNova) aortic valve. Results: All patients presented with multiple comorbidities as reflected by the median EuroSCORE-II of 3.1% (IQR 1.9–5.3). Thirty-four patients from the surgical group (60.7%) underwent a concomitant myocardial revascularization. There was no significant difference in major adverse events, pacemaker implantation or postoperative mortality during follow-up. Both interventions demonstrated technical success with similar mean postoperative pressure gradients at follow-up and no cases of paravalvular leakage. Conclusions: Sutureless aortic valve replacement constitutes a feasible treatment alternative for patients with aortic valve disease who are ineligible for transfemoral access route and/or require concomitant coronary revascularization. With its excellent hemodynamic performance, similar survival compared to TA-TAVR, and high cost-efficiency without compromising the postoperative outcomes and in-hospital length of stay SU-AVR might be considered for patients in the “grey-area” between TAVR and SAVR.

## 1. Introduction

Aortic valve stenosis is the most common heart disease in patients older than 75 years of age [1]. Traditionally, surgical aortic valve replacement (SAVR) has been considered the treatment of choice in patients presenting with severe aortic valve pathologies [2]. Nevertheless, the rapid advancement of transcatheter interventions in the modern era has made transcatheter aortic valve replacement (TAVR) a feasible alternative to conventional SAVR. Moreover, following the release of the PARTNER 3 Trial results, the indications for TAVR in the current European guidelines have been extended to intermediate- and low-risk patients [3,4]. Additionally, several randomized trials have illustrated that due to its non-invasiveness, TAVR performed via transfemoral access is at least non-inferior, if not even superior when compared to SAVR [3,5,6,7,8,9]. Although it would appear that SAVR could be rapidly disappearing from the face of cardiac surgery, giving place to the less invasive TAVR intervention, the reality is still far from it.

While transcatheter methods have emerged as a valuable therapeutic option, TAVR is still not applicable in certain groups of patients for various reasons, including severe peripheral arterial disease, vascular anatomical abnormalities, and concomitant severe coronary artery disease. Therefore, the optimization of SAVR and aortic valve prostheses to achieve even superior results is still required.

Sutureless aortic valve prostheses have been introduced into cardiac surgery quite a while ago with the aim of reducing the cross-clamp and operating times in patients undergoing combined procedures [10]. Moreover, the much simpler implantation technique of sutureless prostheses allows for their broad implementation in minimal invasive aortic valve procedures through a J-sternotomy or right antero-lateral thoracotomy access [11,12]. Due to a considerable number of patients falling into the “grey area” between TAVR and a conventional surgical procedure, several trials have been conducted to compare TAVR with SAVR using rapid deployment or sutureless valve prostheses [13,14,15,16,17,18].

In this study, we sought to compare the early outcomes and hemodynamic performance following transapical TAVR (TA-TAVR) and SAVR with sutureless aortic valve prosthesis (SU-AVR) deploying the Perceval valve (LivaNova). As we aimed to target the group of patients who were ineligible for transfemoral approach, we included only transapical transcatheter procedures into the statistical analysis. Indeed, the latter procedure combines both transcatheter and surgical characteristics and is therefore more appropriate to be compared to SU-AVR. Furthermore, due to our successful institutional implementation of sutureless Perceval valve (LivaNova) for several indications [19], we are not utilizing other rapid deployment aortic valve prostheses.

## 2. Materials and Methods

### 2.1. Study Design and Populations

Between April 2018 and June 2021, 248 consecutive patients underwent either SU-AVR or TA-TAVR at our institution. We analyzed and compared the outcomes and postoperative complications in patients undergoing SU-SAVR using Perceval (Corcym, Saluggia, Italy) or TA-TAVR using the SAPIEN XT™ or SAPIEN 3™ transcatheter heart valve (Edwards Lifesciences, Irvine, CA, USA). Patients were included based upon the requirement for an aortic valve replacement due to either aortic valve stenosis or regurgitation. Patients were excluded if the underlying disease was infective endocarditis of the aortic valve, and if a concomitant valve procedure was required. Data were obtained from the institutional database that includes detailed information on patients’ demographics, baseline clinical characteristics, and their laboratory and hemodynamic parameters, as well as intraoperative variables and postoperative outcomes. Patients were followed up based on information available in their electronic medical records, as well as through telephone interviews. This study conforms to the ethical guidelines of the 1975 Declaration of Helsinki as reflected in a prior approval by the local institutional Ethical Committee (21-10349-BO) and the patients’ individual informed consent was waived.

### 2.2. Study Groups

We stratified the patients according to the operative procedures into two groups. Group A included patients who underwent a SU-AVR (*n* = 79), and Group B included patients after TA-TAVR (*n* = 169). The patients were matched based on the EuroSCORE II, resulting in 56 well-balanced matched pairs which were analyzed. The patients who underwent a concomitant mitral- or tricuspid valve procedure were excluded from the study.

### 2.3. Operative Techniques

All transcatheter procedures were performed via transapical access, under general anesthesia, and in the presence of our institutional Heart Team working in a special equipped hybrid operating theatre [20]. The TA-TAVR technique deployed was previously described [21].

For SU-AVR, the heart was accessed via median sternotomy. Cardiopulmonary bypass (CPB) was initiated with a direct cannulation of the ascending aorta and cannulation of the right atrium. Moderate hypothermic cardiac arrest at 35 °C was performed in all procedures. Myocardial protection was achieved with cold crystalloid cardioplegia. The aortic valve was exposed and excised through an oblique aortotomy. The implantation of sutureless Perceval prostheses was performed using the snugger method as described previously [10,22], and the aortotomy was closed with a 4.0 prolene suture. After assessment of the valve performance and careful de-airing, the patient was weaned from the CPB.

The closure of patent foramen ovale (PFO) and MAZE procedure were performed on CBP on the beating heart, and coronary arterial bypass grafting (CABG) was performed on the arrested heart prior to the SU-AVR. Proximal coronary anastomoses were performed on the arrested heart to avoid additional manipulation of the aorta after the sutureless valve prosthesis had been implanted.

### 2.4. Outcomes and Definitions

The primary endpoints were 30-day mortality and 6-month mortality. The secondary endpoint was the development of any complications according to the Valve Academic Research Consortium-2 [23]. Urgent procedures were defined as procedures which had to be performed in the same in-hospital stay. Emergent procedures were defined as procedures which had to be performed within the next 24 h. A major adverse event, as defined by the FDA, is any untoward clinical occurrence which results in death of the patient, is life-threatening, causes a significant prolongation of existing hospitalization, results in significant disability/incapacity, or requires intervention to prevent a permanent impairment or damage.

### 2.5. Statistical Analysis

Statistical analysis was performed using IBM SPSS version 27 (IBM Corp., Chicago, IL, USA) and R software v.3.4.3 (R Foundation for Statistical Computing, Vienna, Austria). Data were tested for normality using the Shapiro-Wilk test. Continuous variables were expressed as medians (interquartile range, IQR) or as mean ± standard deviation. Categorical variables were expressed as frequencies and percentages. We compared the distributions of the categorical variables using the Chi-square test or Fischer exact test if the assumptions for the first one were not met. The distributions of the continuous variables were compared between the groups with the t-test in cases of normal distributions and with the Mann–Whitney test if the distributions were not normal. A *p*-value of less than 0.05 was considered to indicate statistical significance. For plotting the survival curves and for computing the mid-term mortality, we used the Kaplan–Meier method. The cumulative survivals of both methods were analyzed and compared with the log rank test.

## 3. Results

### 3.1. Baseline Characteristics

The mean age of the patients was 71.6 ± 8.2 years (Table 1). All patients presented with symptomatic moderate-to-severe aortic stenosis (*n* = 108, 96.4%) and/or moderate-to-severe aortic regurgitation (*n* = 10, 8.9%), and were suffering from dyspnea (NYHA Class I, II and III *n* = 18 (16.1%), *n* = 54 (48.2%) and *n* = 41 (36.6%), respectively). Significantly more patients in the surgical group presented with moderate-to-severe aortic regurgitation (*p* = 0.001). Patients presented with multiple comorbidities as reflected by the median logistic EuroSCORE of 6.9% (IQR 3.6–13.0) and a median EuroSCORE-II of 3.1% (IQR 1.9–5.3). Severe pulmonary hypertension was present in 8.9% of the patients (*n* = 10) and 85 patients (75.9%) were suffering from coronary artery disease.

The patients in the surgical arm were slightly younger than the patients in the transcatheter arm (69.1 ± 7.9 vs. 74.3 ± 7.8 years old respectively, *p* = 0.001). Additionally, the patients in the transcatheter group were significantly more likely to suffer from peripheral artery disease (*p* < 0.001).

### 3.2. Intraoperative Characteristics

Out of all the patients, 21 (18.8%) underwent an urgent procedure and 3 patients (2.7%) underwent an emergent operation (Table 2). There was no significant difference between the two groups regarding the urgency of the procedure. Thirty-four patients in the surgical group (60.7%) underwent a concomitant CABG procedure. Of the whole cohort, six patients (5.4%) had previously undergone a cardiac procedure via median sternotomy. Operating time was significantly higher in the surgical arm when compared to the transcatheter group (149.1 ± 48.3 min vs. 67.3 ± 34.7 min respectively, *p* < 0.001). The patients in the surgical group needed significantly more blood transfusions than the transcatheter group (*p* < 0.001).

### 3.3. Postoperative Characteristics and Survival

**Figure 1:**
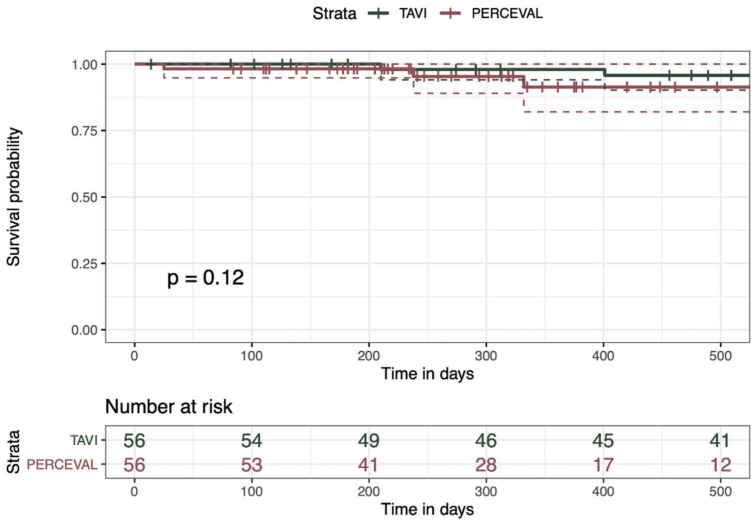
Survival of patients undergoing SU-AVR and TA-TAVR.

The mean follow-up time was 18.1 ± 12.3 months. There was no significant difference in the major adverse events, pacemaker implantation or postoperative mortality during the follow-up between the two groups (Table 3, Figure 1). Additionally, the mean postoperative pressure gradient at follow-up was similar between the groups (6.0 mmHg (IQR 4.25–7.0) in SU-SAVR vs. 5.0 mmHg (IQR 4.0–6.0) in TA-TAVR, *p* = 0.125). None of the patients in the whole cohort presented with paravalvular leakage. There was no difference in the rate of postoperative aortic valve regurgitation leakage between the groups.

### 3.4. Figures and Tables

The figure presents the survivals of patients undergoing a SU-AVR and TA-TAVI (transcatheter aortic valve implantation) presented with Kaplan—Meier curves. The survival rates were analyzed and compared with the log rank test and show no statistical difference (*p* > 0.05).

## 4. Discussion

In the present study, a total of 112 intermediate-risk [24] patients presenting with moderate-to severe aortic valve disease were treated either by conventional SAVR with sutureless valve prosthesis or TA-TAVR. This study provides a number of interesting findings:

1. SU-AVR is a feasible and safe treatment option that presents itself as at least a non-inferior alternative to TA-TAVR in intermediate-risk patients.
2. Both treatments offer a high technical procedural success, nonetheless, SU-AVR offers significantly longer operating times with no negative effect on the length of stay at the intensive care unit and ventilation time.
3. Both treatment options present no significant difference in postoperative pacemaker implantation and stroke.
4. Despite the use of contrast medium in the TA-TAVI group, there was no significant difference in postoperative new onset dialysis rate and maximum creatinine levels when compared to SU-AVR.
5. Patients from the surgical arm needed significantly more intraoperative blood transfusions.
6. There was no significant difference in the hemodynamic performance of the valve prostheses between the groups. Both methods provided low postoperative transvalvular gradients with no increased risk of paravalvular leakage in both groups.
7. There was no significant difference in 30-day and 6-month mortality.

Following the great successes of transcatheter technologies in aortic valve replacement in high-risk patients cohorts, TAVR procedures were also recently implemented as an alternative to conventional surgical aortic valve replacement in intermediate- and low-risk patients [4]. Although the most common transcatheter access is transfemoral, there is still a portion of patients who are not eligible for this approach. These patients are considered to be in the “grey-area” between the transfemoral TAVR and conventional SAVR and could benefit from any other alternative to both procedures. Whilst transapical TAVR access is a standard route for TAVR in patients ineligible for transfemoral access, SU-AVR proved to be a feasible alternative for TAVR in patients with intermediate or high surgical risk [1,18,21,25]. Therefore, hereby we sought to evaluate our results with SU-AVR in intermediate-risk patients and compare them to those of TA-TAVR.

In the following study, we performed a pair-matched analysis based on the EuroSCORE II. Both groups included 56 patients and showed to be well balanced and comparable with regards to preoperative characteristics, although the surgical procedure performed on the CPB with cardiac arrest tends to carry higher operative risk in terms of bleeding, blood transfusion, and longer procedural time (Table 1). Nonetheless, the core preoperative parameters, which are considered to be responsible for the higher risk score, are included into the EuroSCORE II calculating protocol, thereby making this parameter suitable for pair matching.

In our study, we found that SU-AVR is a feasible and safe treatment option that offers a non-inferior alternative to TA-TAVR in intermediate-risk patients. Thirty-day and six-month mortality was similar between the groups. Furthermore, 30-day mortality in the surgical arm (1.8%) was illustrated to be comparable with the results of the international registry (SURD-IR), which in turn reported an in-hospital mortality of 2.1% in patients undergoing a SAVR with sutureless- or rapid deployment aortic valve prostheses [26,27]. In our SU-AVR cohort, the implementation of the sutureless prostheses was chosen in order to save the cross-clamp time in consideration of the patients’ multiple comorbidities and the need of a concomitant procedure. Moreover, 34 patients (60.7%) in the surgical arm required a concomitant CABG procedure, while 42 patients (75.0%) in the TA-TAVR group suffering from coronary artery disease had been treated or were planned for a staged approach treatment. Concomitant treatment of CAD and transcatheter treatment of severe aortic stenosis is a current matter of debate and there is still no clear treatment strategy [28].

Although patients in the surgical group underwent a procedure on CPB with cardiac arrest, the significantly longer procedural time compared to the transcatheter group was not associated with increased mortality, as previously also stated in the study by Swinkels et al. [29].

One of the most feared complications after cardiothoracic procedures is a disabling stroke. In the SURD-IR registry, the authors reported a stroke rate of 2.8% in patients undergoing isolated SAVR with sutureless or rapid deployment valve prostheses. In contrast to other studies, in our cohort, we observed a very low stroke rate in the transcatheter group (1.8%) compared to the results of the PARTNER 1 trial, and none in the surgical group (Table 3) [30]. This could be explained by the technical specifications of the procedures we carried out. Indeed, all the proximal CABG anastomoses were performed under cardiac arrest without partial clamping of the aorta to reduce the manipulation and dislocation risk of the Perceval prosthesis. Additionally, during the transcatheter procedures, the direct antegrade placement of the guiding wire prevents from excessively manipulating the inner wall of a possible calcified aorta.

According to our previously published modified implantation height, we observed just one case of postprocedural pacemaker implantation (1.8%) in the transcatheter cohort, which is considerably lower when compared to the 6.5% reported in the PARTNER3 trial [3,31]. Moreover, based on our experience with the 3rd generation TAVR devices, this implantation technique provides postoperative pacemaker implantation rates of less than 5% [32,33]. Although the SURD-IR registry described a postoperative pacemaker implantation rate of 5.4%, in the surgical arm of our cohort, we observed no cases of postoperative pacemaker implantation. The continuous reduction in postoperative pacemaker implantation risk over the years described in the SURD-IR registry is also reflected in the results and experience of our team throughout our years of experience. Indeed, our previously analyzed cohort of patients undergoing SU-AVR reported a risk of postoperative pacemaker implantation of 3.1% [19].

Furthermore, in the present study, there was no significant difference in the preoperative kidney function between the groups (Table 1). Kidney function has already been described as an independent predictor of outcome in patients undergoing transcatheter valve procedures [34]. Although all TA-TAVR procedures were performed with contrast dye application, it still had no significant impact the postoperative kidney function and new postoperative dialysis rate (Table 3). To further reduce the amount of intraoperative contrast use, we used a pigtail catheter within the non-coronary sinus as an additional reference marker during the implantation [21].

In accordance with the findings of previous studies, we could show that both procedures provide comparable and excellent hemodynamic results with low transvalvular gradients at follow-up [21,27]. Follow-up echocardiography showed mean transvalvular gradients in the transcatheter arm of 5.0 mmHg (IQR 4.0–6.0), compared to 6.0 mmHg (IQ 4.25–7.0) in the surgical group.

As the SU-AVR is obviously a more invasive procedure than TA-TAVR, requiring a sternotomy and heparin application for the CPB, it is not surprising that patients in the surgical arm needed significantly more blood transfusions. Nonetheless, despite the results reported by Bjursten et al., the higher rate of blood transfusions does not seem to negatively impact the outcomes in the surgical group of our cohort [35].

## 5. Conclusions

Whilst indication for transcatheter aortic valve replacement has been extended toward intermediate- and low-risk patients, there is still a portion of patients who are not eligible for transfemoral access or require a concomitant myocardial revascularization. For a long time, transapical transcatheter aortic valve replacement and staged percutaneous coronary intervention has been a treatment option for those patients. Surgical procedures with reduced invasiveness and novel superior valve prostheses have been implemented in cardiac surgery to stand the competition with transcatheter methods. Sutureless aortic valve replacement offers a feasible treatment alternative for patients with aortic valve disease who are ineligible for transfemoral access route and/or need a concomitant coronary revascularization. SU-AVR offers an excellent hemodynamic performance and similar survival, compared to TA-TAVR. Additionally, SU-AVR comes with a high cost efficiency without compromising the postoperative outcomes and in-hospital length of stay.

### Study Limitations

The retrospective non-randomized nature of the study coming from a single center with a limited number of patients may have an impact on the outcomes and the study power, and can leave room for bias. Additionally, we did not take into consideration other alternative TAVR access routes as trans-axillary or trans-carotid access, as both methods are not yet widely used at our institution. Unfortunately, there are only few studies with small cohorts which compare SU-AVR and TA-TAVR procedures. Further prospective studies on larger cohorts should be conducted to validate the safety and efficiency of this therapeutic alternative.

## Figures and Tables

**Table 1:** Baseline characteristics.

Characteristics	All Patients (*n* = 112)	TA-TAVR (*n* = 56)	SURD-AVR (*n* = 56)	*p*-Value
female sex	27 (24.1%)	14 (25%)	13 (23.2%)	0.825
age, years	71.6 ± 8.2	74.3 ± 7.8	69.05 ± 7.9	0.001
BMI, kg/qcm	28.8 ± 6.5	28.8 ± 7.6	28.8 ± 5.4	0.322
arterial hypertension	106 (94.6%)	52 (92.9%)	54 (96.4%)	0.401
pulmonary hypertension	10 (8.9%)	4 (7.1%)	6 (10.7%)	0.508
hyperlipidemia	61 (54.5%)	28 (50.0%)	33 (58.9%)	0.343
renal insufficiency	30 (26.8%)	17 (30.4%)	13 (23.2%)	0.393
previous MI	23 (20.5%)	15 (26.8%)	8 (14.3%)	0.101
COPD	26 (23.2%)	15 (26.8%)	11 (19.6%)	0.371
CAD	85 (75.9%)	42 (75.0%)	43 (76.8%)	0.825
PAD	38 (33.9%)	29 (51.8%)	9 (16.1%)	<0.001
NYHA Class				
I	18 (16.1%)	14 (25%)	4 (7.1%)	0.01
II	54 (48.2%)	21 (37.5%)	33 (58.9%)	0.023
III	41 (36.6%)	21 (37.5%)	20 (35.7%)	0.844
creatinine, mg/dL	1.4 ± 1.3	1.4 ± 1.1	1.4 ± 1.4	0.4
bilirubine, mg/dL	0.7 ± 0.6	0.6 ± 0.4	0.76 ± 0.8	0.79
diabetes mellitus type 2	39 (34.8%)	20 (35.7%)	19 (33.9%)	0.843
dialysis	9 (8%)	5 (8.9%)	4 (7.1%)	1.0
EF, %	51.6 ± 9.5	51.9 ± 9.3	51.2 ± 9.9	0.676
AS > II	108 (96.4%)	56 (100%)	52 (92.9%)	0.042
AR > II	10 (8.9%)	0	10 (17.9%)	0.001
logistic EuroSCORE I	6.9 (IQR 3.6–13.0)	8.0 (IQR 4.5–13.0)	5.4 (IQR 2.9–13.2)	0.18
EuroSCORE II	3.1 (IQR 1.9–5.3)	3.2 (IQR 2.3–5.4)	2.8 (IQR 1.5–5.2)	0.112

AR—aortic regurgitation, AS—aortic stenosis, BMI—body mass index, CAD—coronary arterial disease, COPD—chronic obstructive pulmonary disease, EF—ejection fraction, MI—myocardial infarction, NYHA—New York Heart Association Class, PAD—peripheral arterial disease. Continuous variables were expressed as medians (interquartile range, IQR) or as mean ± standard deviation.

**Table 2:** Intraoperative characteristics.

Characteristics	All Patients (*n* = 112)	TA-TAVR (*n* = 56)	SURD-AVR (*n* = 56)	*p*-Value
elective	87 (77.7%)	45 (80.4%)	42 (75.0%)	0.496
urgent	21 (18.8%)	10 (17.9%)	11 (19.6%)	0.809
emergent	3 (2.7%)	1 (1.8%)	2 (3.6%)	1.0
redo	6 (5.4%)	1 (1.8%)	5 (8.9%)	0.206
concomitant procedure	36 (32.1%)	-	36 (64.3%)	-
CABG	34 (30.4%)	-	34 (60.7%)	-
MAZE	3 (2.7%)	-	3 (5.4%)	-
PFO closure	2 (1.8%)	-	2 (3.6%)	-
operating time, min	108.2 ± 58.6	67.3 ± 34.7	149.05 ± 48.3	<0.001
cross-clamp time, min	9.0 (IQR 0.0–47.0)	-	49.4 ± 17.4	-
blood transfusion	37 (33.0%)	6 (10.7%)	31 (55.4%)	<0.001
Erythrocytes, Units	0.0 (IQR 0.0–2.0)	0.0 (IQR 0.0–0.0)	1.0 (IQR 0.0–2.0)	<0.001
Perceval				
S	8 (7.1%)	-	8 (14.3%)	-
M	11 (9.8%)	-	11 (19.6%)	-
L	16 (14.3%)	-	16 (28.6%)	-
XL	22 (19.6%)	-	22 (39.2%)	-
Sapien				
23	18 (16.1%)	18 (32.2%)	-	-
26	28 (25%)	28 (50.0%)	-	-
29	9 (8%)	9 (16%)	-	-
contrast dye, ml	-	75 (IQR 50.0–93.75)	-	-

CABG—coronary arterial bypass grafting, PFO—patent foramen ovale. Continuous variables were expressed as medians (interquartile range, IQR) or as mean ± standard deviation.

**Table 3:** Postoperative characteristics.

Characteristics	All Patients (*n* = 112)	TA-TAVR (*n* = 56)	SURD-AVR (*n* = 56)	*p*-Value
stroke	1 (0.9%)	1 (1.8%)	0	1.0
maximal creatinine, mg/dl	1.23 (IQR 1.0–1.9)	1.2 (IQR 1.0–1.8)	1.25 (IQR 0.9–1.9)	0.86
maximal biliribine, mg/dl	0.85 (IQR 0.6–1.3)	0.8 (IQR 0.6–1.2)	0.9 (IQR 0.7–1.7)	0.07
pacemaker implantation	1 (0.9%)	1 (1.8%)	0	1.0
new dialysis	8 (7.1%)	3 (5.4%)	5 (8.9%)	0.72
mechanical ventilation, days	1.0 (IQR 1.0–1.0)	1.0 (IQR 1.0–1.0)	1.0 (IQR 1.0–1.75)	0.42
re-intubation	1 (0.9%)	0	1 (1.8%)	1.0
Low output syndrome	4 (3.6%)	1 (1.8%)	3 (5.4%)	0.61
AR at discharge	0	0	0	-
re-thoracotomy	6 (5.4%)	0	6 (10.7%)	0.027
wound infection	3 (2.7%)	0	3 (5.4%)	0.243
postoperative MPG, mmHg	5.7 ± 1.7	5.0 (IQR 4.0–6.0)	6.0 (IQR 4.25–7.0)	0.125
hospital LOS, days	8.9 ± 4.5	8.2 ± 3.4	9.7 ± 5.3	0.25
ICU LOS, days	2.0 (IQR 1.0–4.0)	2.0 (IQR 2.0–4.0)	2.0 (IQR 1.0–3.75)	0.39
follow-up, months	18.1 ± 12.3	25.1 ± 12.8	11.0 ± 6.2	<0.001
30-day mortality	1 (0.9%)	0	1 (1.8%)	1.0
6-month mortality	1 (0.9%)	0	1 (1.8%)	1.0

AR—aortic regurgitation, ICU—intensive care unit, LOS—length of stay, MPG—mean pressure gradient. Continuous variables were expressed as medians (interquartile range, IQR) or as mean ± standard deviation.

## Data Availability

Data available on request from the authors. The data that support the findings of this study are available from the corresponding author upon reasonable request. (E-Mail: alina.zubarevich@gmail.com).

## References

[1] Meco M., Miceli A., Montisci A., Donatelli F., Cirri S., Ferrarini M., Lio A., Glauber M. (2017). Sutureless aortic valve replacement versus transcatheter aortic valve implantation: A meta-analysis of comparative matched studies using propensity score matching. Interact. Cardiovasc. Thorac. Surg..

[2] Baumgartner H., Falk V., Bax J.J., De Bonis M., Hamm C., Holm P.J., Iung B., Lancellotti P., Lansac E., Rodriguez Muñoz D. (2017). 2017 ESC/EACTS Guidelines for the management of valvular heart disease. Eur. J. Cardio-Thorac. Surg..

[3] Mack M.J., Leon M.B., Thourani V.H., Makkar R., Kodali S.K., Russo M. (2019). Transcatheter aortic-valve replacement with a bal-loon-expandable valve in low-risk patients. N. Engl. J. Med..

[4] Beyersdorf F., Vahanian A., Milojevic M., Praz F., Baldus S., Bauersachs J., Capodanno D., Conradi L., De Bonis M., De Paulis R. (2022). Corrigendum to: 2021 ESC/EACTS Guidelines for the management of valvular heart disease. Eur. J. Cardio-Thoracic Surg..

[5] Smith C.R., Leon M.B., Mack M.J., Miller D.C., Moses J.W., Svensson L.G., Tuzcu E.M., Webb J.G., Fontana G.P., Makkar R.R. (2011). Transcatheter versus Surgical Aortic-Valve Replacement in High-Risk Patients. N. Engl. J. Med..

[6] Thourani V.H., Kodali S., Makkar R.R., Herrmann H.C., Williams M., Babaliaros V., Smalling R., Lim S., Malaisrie S.C., Kapadia S. (2016). Transcatheter aortic valve replacement versus surgical valve replacement in intermediate-risk patients: A propensity score analysis. Lancet.

[7] Mack M.J., Leon M.B., Smith C.R., Miller D.C., Moses J.W., Tuzcu E.M., Webb J.G., Douglas P.S., Anderson W.N., Blackstone E.H. (2015). 5-year outcomes of transcatheter aortic valve replacement or surgical aortic valve replacement for high surgical risk patients with aortic stenosis (PARTNER 1): A randomised controlled trial. Lancet.

[8] Leon M.B., Smith C.R., Mack M.J., Makkar R.R., Svensson L.G., Kodali S.K., Thourani V.H., Tuzcu E.M., Miller D.C., Herrmann H.C. (2016). Transcatheter or Surgical Aortic-Valve Replacement in Intermediate-Risk Patients. N. Engl. J. Med..

[9] Reardon M.J., Heijmen R.H., Van Mieghem N.M., Williams M.R., Yakubov S.J., Watson D., Kleiman N.S., Conte J., Chaawla A., Hockmth D. (2019). Comparison of Outcomes after Transcatheter vs Surgical Aortic Valve Replacement among Patients at Intermediate Operative Risk with a History of Cor-onary Artery Bypass Graft Surgery: A Post Hoc Analysis of the SURTAVI Randomized Clinical Trial. JAMA Cardiol..

[10] Zubarevich A., Szczechowicz M., Zhigalov K., Osswald A., Eynde J.V.D., Rad A.A., Vardanyan R., Wendt D., Schmack B., Ruhparwar A. (2021). Sutureless aortic valve replacement in multivalve procedures. J. Thorac. Dis..

[11] Di Eusanio M., Vessella W., Carozza R., Capestro F., D’Alfonso A., Zingaro C., Munch C., Berretta P. (2018). Ultra fast-track minimally invasive aortic valve replacement: Going beyond reduced incisions. Eur. J. Cardio Thorac. Surg. Off. J. Eur. Assoc. Cardio Thorac. Surg..

[12] Zubarevich A., Zhigalov K., Schmack B., Rad A.A., Vardanyan R., Wendt D., Ruhparwar A., Weymann A. (2021). Step-by-Step Minimally Invasive Aortic Valve Replacement: The RAT Approach. Braz. J. Cardiovasc. Surg..

[13] D’Onofrio A., Messina A., Lorusso R., Alfieri O.R., Fusari M., Rubino P., Rinaldi M., Di Bartolomeo R., Glauber M., Troise G. (2012). Sutureless aortic valve replacement as an alternative treatment for patients belonging to the “gray zone” between transcatheter aortic valve implantation and conventional surgery: A propensity-matched, multicenter analysis. J. Thorac. Cardiovasc. Surg..

[14] Santarpino G., Pollari F., Fischlein T. (2014). Sutureless versus transcatheter aortic valve implantation: An unresolved dilemma. J. Thorac. Cardiovasc. Surg..

[15] Santarpino G., Pfeiffer S., Jessl J., Dell’Aquila A.M., Pollari F., Pauschinger M., Fischlein T. (2014). Sutureless replacement versus transcatheter valve implantation in aortic valve stenosis: A propensity-matched analysis of 2 strategies in high-risk patients. J. Thorac. Cardiovasc. Surg..

[16] Repossini A., Fischlein T., Solinas M., DIBacco L., Passaretti B., Grubitzsch H., Santarpino F.T., di Bartolomeo R., Laborde F., Muneretto C. (2018). Stentless sutureless and transcatheter valves: A comparison of the hemodynamic performance of different prostheses concept. Minerva Cardioangiol..

[17] Takagi H., Umemoto T. (2015). Sutureless aortic valve replacement may improve early mortality compared with transcatheter aortic valve implantation: A meta-analysis of comparative studies. J. Cardiol..

[18] Al-Maisary S., Farag M., Gussinklo W.T., Kremer J., Pleger S., Leuschner F., Karck M., Szabo G., Arif R. (2021). Are Sutureless and Rapid-Deployment Aortic Valves a Serious Alternative to TA-TAVI? A Matched-Pairs Analysis. J. Clin. Med..

[19] Mashhour A., Zhigalov K., Mkalaluh S., Szczechowicz M., Easo J., Eichstaedt H., Weymann A. (2020). Outcome of a Modified Perceval Implan-tation Technique. Thorac. Cardiovasc. Surg..

[20] Zubarevich A., Szczechowicz M., Rad A.A., Vardanyan R., Marx P., Lind A., Jánosi R.A., Roosta-Azad M., Malik R., Kamler M. (2021). Mitral surgical redo versus transapical transcatheter mitral valve implantation. PLoS ONE.

[21] Zubarevich A., Szczechowicz M., Jánosi R.-A., Lind A., Rassaf T., Malik R., Thielmann M., Schmack B., Kamler M., Ruhparwar A. (2021). Alternative access in high-risk patients in the era of transfemoral aortic valve replacement. Minim. Invasive Ther. Allied Technol..

[22] Mashhour A., Zhigalov K., Szczechowicz M., Mkalaluh S., Easo J., Eichstaedt H., Borodin D., Ennker J., Weymann A. (2018). Snugger method—The Oldenburg modi-fication of perceval implantation technique. World J Cardiol..

[23] Kappetein A.P., Head S.J., Généreux P., Piazza N., van Mieghem N.M., Blackstone E.H., Brott T.G., Cogen D.J., Cutlip D.E., van Es G.-A. (2013). Updated standardized endpoint def-initions for transcatheter aortic valve implantation: The Valve Academic Research Consortium-2 consensus document. J. Thorac. Cardiovasc. Surg..

[24] Burke L., Gozdecki L., Doukas D., Joyce C., Weaver F., A Bavry A., Garcia S., Cohen D.J., A Shunk K., Mathew V. (2020). Patient Risk Assessment for Transcatheter Aortic Valve Replacement at Veterans Health Administration Hospitals. J. Invasive Cardiol..

[25] Muneretto C., Bisleri G., Moggi A., Di Bacco L., Tespili M., Repossini A., Rambaldini M. (2015). Treating the patients in the “grey-zone” with aortic valve disease: A comparison among conventional surgery, sutureless valves and transcatheter aortic valve replacement. Interact. Cardiovasc. Thorac. Surg..

[26] Berretta P., Meuris B., Kappert U., Andreas M., Fiore A., Solinas M., Misfeld M., Carrel T.P., Villa E., Sacini C. (2021). Sutureless Versus Rapid Deployment Aortic Valve Replacement: Results from a Multicenter Registry. Ann. Thorac. Surg.

[27] Di Eusanio M., Phan K., Berretta P., Carrel T.P., Andreas M., Santarpino G., di Bartolomeo R., Folliguet T., Meuris B., Mignosa C. (2018). Sutureless and Rapid-Deployment Aortic Valve Replacement International Registry (SURD-IR): Early results from 3343 patients. Eur J. Cardiol. Thorac. Surg. Off. J. Eur. Assoc. Cardiol. Thorac. Surg..

[28] Zubarevich A., Kadyraliev B., Arutyunyan V., Chragyan V., Askadinov M., Sozkov A., Ponomarev D., Zyazeva I., Sá M.P.B.O., Osswald A. (2020). On-pump versus off-pump coronary artery bypass surgery for multi-vessel coronary revascularization. J. Thorac. Dis..

[29] Swinkels B.M., Berg J.M.T., Kelder J.C., Vermeulen F.E., Van Boven W.J., A de Mol B. (2020). Effect of aortic cross-clamp time on late survival after isolated aortic valve replacement. Interact. Cardiovasc. Thorac. Surg..

[30] Kapadia S., Agarwal S., Miller D.C., Webb J.G., Mack M., Ellis S., Herrmann H.C., Pichard A.D., Tuzcu E.M.T., Svensson L.G. (2016). Insights Into Timing, Risk Factors, and Outcomes of Stroke and Transient Ischemic Attack After Transcatheter Aortic Valve Replacement in the PARTNER Trial (Placement of Aortic Transcatheter Valves). Circ. Cardiovasc. Interv..

[31] Wendt D., Al-Rashid F., Kahlert P., Eißmann M., El-Chilali K., Jánosi R.A., Pasa S., Tsagakis K., Liakopoulos O., Erbel R. (2015). Low Incidence of Paravalvular Leakage with the Balloon-Expandable Sapien 3 Transcatheter Heart Valve. Ann. Thorac. Surg..

[32] Wendt D., Shehada S.-E., König L., Kahlert P., Frey U., Mourad F., Jakob H., Thielmann M., El Gabry M. (2020). Modified implantation height of the Sapien3TM transcatheter heart valve. Off. J. Soc. Minim. Invasive Ther..

[33] Schwerg M., Fulde F., Dreger H., Poller W.C., Stangl K., Laule M. (2016). Optimized Implantation Height of the Edwards SAPIEN 3 Valve to Minimize Pacemaker Implantation after TAVI. J. Interv. Cardiol..

[34] Lanz J., Kim W.-K., Walther T., Burgdorf C., Möllmann H., Linke A., Redwood S., Thilo C., Hilker M., Joner M. (2019). Safety and efficacy of a self-expanding versus a bal-loon-expandable bioprosthesis for transcatheter aortic valve replacement in patients with symptomatic severe aortic ste-nosis: A randomised non-inferiority trial. Lancet.

[35] Bjursten H., Al-Rashidi F., Dardashti A., Brondén B., Algotsson L., Ederoth P. (2013). Risks Associated with the Transfusion of Various Blood Products in Aortic Valve Replacement. Ann. Thorac. Surg..

